# Measurements of triple-differential cross sections for inclusive isolated-photon+jet events in $\mathrm{p}\mathrm{p}$ collisions at $\sqrt{s} = 8\,\text {TeV}$

**DOI:** 10.1140/epjc/s10052-019-7451-7

**Published:** 2019-11-25

**Authors:** A. M. Sirunyan, A. Tumasyan, W. Adam, F. Ambrogi, E. Asilar, T. Bergauer, J. Brandstetter, M. Dragicevic, J. Erö, A. Escalante Del Valle, M. Flechl, R. Frühwirth, V. M. Ghete, J. Hrubec, M. Jeitler, N. Krammer, I. Krätschmer, D. Liko, T. Madlener, I. Mikulec, N. Rad, H. Rohringer, J. Schieck, R. Schöfbeck, M. Spanring, D. Spitzbart, W. Waltenberger, J. Wittmann, C.-E. Wulz, M. Zarucki, V. Chekhovsky, V. Mossolov, J. Suarez Gonzalez, E. A. De Wolf, D. Di Croce, X. Janssen, J. Lauwers, A. Lelek, M. Pieters, H. Van Haevermaet, P. Van Mechelen, N. Van Remortel, S. Abu Zeid, F. Blekman, J. D’Hondt, J. De Clercq, K. Deroover, G. Flouris, D. Lontkovskyi, S. Lowette, I. Marchesini, S. Moortgat, L. Moreels, Q. Python, K. Skovpen, S. Tavernier, W. Van Doninck, P. Van Mulders, I. Van Parijs, D. Beghin, B. Bilin, H. Brun, B. Clerbaux, G. De Lentdecker, H. Delannoy, B. Dorney, G. Fasanella, L. Favart, R. Goldouzian, A. Grebenyuk, A. K. Kalsi, T. Lenzi, J. Luetic, N. Postiau, E. Starling, L. Thomas, C. Vander Velde, P. Vanlaer, D. Vannerom, Q. Wang, T. Cornelis, D. Dobur, A. Fagot, M. Gul, I. Khvastunov, D. Poyraz, C. Roskas, D. Trocino, M. Tytgat, W. Verbeke, B. Vermassen, M. Vit, N. Zaganidis, H. Bakhshiansohi, O. Bondu, S. Brochet, G. Bruno, C. Caputo, P. David, C. Delaere, M. Delcourt, A. Giammanco, G. Krintiras, V. Lemaitre, A. Magitteri, K. Piotrzkowski, A. Saggio, M. Vidal Marono, P. Vischia, S. Wertz, J. Zobec, F. L. Alves, G. A. Alves, G. Correia Silva, C. Hensel, A. Moraes, M. E. Pol, P. Rebello Teles, E. Belchior Batista Das Chagas, W. Carvalho, J. Chinellato, E. Coelho, E. M. Da Costa, G. G. Da Silveira, D. De Jesus Damiao, C. De Oliveira Martins, S. Fonseca De Souza, H. Malbouisson, D. Matos Figueiredo, M. Melo De Almeida, C. Mora Herrera, L. Mundim, H. Nogima, W. L. Prado Da Silva, L. J. Sanchez Rosas, A. Santoro, A. Sznajder, M. Thiel, E. J. Tonelli Manganote, F. Torres Da Silva De Araujo, A. Vilela Pereira, S. Ahuja, C. A. Bernardes, L. Calligaris, T. R. Fernandez Perez Tomei, E. M. Gregores, P. G. Mercadante, S. F. Novaes, SandraS. Padula, A. Aleksandrov, R. Hadjiiska, P. Iaydjiev, A. Marinov, M. Misheva, M. Rodozov, M. Shopova, G. Sultanov, A. Dimitrov, L. Litov, B. Pavlov, P. Petkov, W. Fang, X. Gao, L. Yuan, M. Ahmad, J. G. Bian, G. M. Chen, H. S. Chen, M. Chen, Y. Chen, C. H. Jiang, D. Leggat, H. Liao, Z. Liu, S. M. Shaheen, A. Spiezia, J. Tao, E. Yazgan, H. Zhang, S. Zhang, J. Zhao, Y. Ban, G. Chen, A. Levin, J. Li, L. Li, Q. Li, Y. Mao, S. J. Qian, D. Wang, Y. Wang, C. Avila, A. Cabrera, C. A. Carrillo Montoya, L. F. Chaparro Sierra, C. Florez, C. F. González Hernández, M. A. Segura Delgado, B. Courbon, N. Godinovic, D. Lelas, I. Puljak, T. Sculac, Z. Antunovic, M. Kovac, V. Brigljevic, D. Ferencek, K. Kadija, B. Mesic, M. Roguljic, A. Starodumov, T. Susa, M. W. Ather, A. Attikis, M. Kolosova, G. Mavromanolakis, J. Mousa, C. Nicolaou, F. Ptochos, P. A. Razis, H. Rykaczewski, M. Finger, M. Finger, E. Ayala, E. Carrera Jarrin, M. A. Mahmoud, A. Mahrous, Y. Mohammed, S. Bhowmik, A. Carvalho Antunes De Oliveira, R. K. Dewanjee, K. Ehataht, M. Kadastik, M. Raidal, C. Veelken, P. Eerola, H. Kirschenmann, J. Pekkanen, M. Voutilainen, J. Havukainen, J. K. Heikkilä, T. Järvinen, V. Karimäki, R. Kinnunen, T. Lampén, K. Lassila-Perini, S. Laurila, S. Lehti, T. Lindén, P. Luukka, T. Mäenpää, H. Siikonen, E. Tuominen, J. Tuominiemi, T. Tuuva, M. Besancon, F. Couderc, M. Dejardin, D. Denegri, J. L. Faure, F. Ferri, S. Ganjour, A. Givernaud, P. Gras, G. Hamel de Monchenault, P. Jarry, C. Leloup, E. Locci, J. Malcles, G. Negro, J. Rander, A. Rosowsky, M. Ö. Sahin, M. Titov, A. Abdulsalam, C. Amendola, I. Antropov, F. Beaudette, P. Busson, C. Charlot, R. Granier de Cassagnac, I. Kucher, A. Lobanov, J. Martin Blanco, C. Martin Perez, M. Nguyen, C. Ochando, G. Ortona, P. Paganini, J. Rembser, R. Salerno, J. B. Sauvan, Y. Sirois, A. G. Stahl Leiton, A. Zabi, A. Zghiche, J.-L. Agram, J. Andrea, D. Bloch, J.-M. Brom, E. C. Chabert, V. Cherepanov, C. Collard, E. Conte, J.-C. Fontaine, D. Gelé, U. Goerlach, M. Jansová, A.-C. Le Bihan, N. Tonon, P. Van Hove, S. Gadrat, S. Beauceron, C. Bernet, G. Boudoul, N. Chanon, R. Chierici, D. Contardo, P. Depasse, H. El Mamouni, J. Fay, L. Finco, S. Gascon, M. Gouzevitch, G. Grenier, B. Ille, F. Lagarde, I. B. Laktineh, H. Lattaud, M. Lethuillier, L. Mirabito, S. Perries, A. Popov, V. Sordini, G. Touquet, M. Vander Donckt, S. Viret, T. Toriashvili, I. Bagaturia, C. Autermann, L. Feld, M. K. Kiesel, K. Klein, M. Lipinski, M. Preuten, M. P. Rauch, C. Schomakers, J. Schulz, M. Teroerde, B. Wittmer, A. Albert, D. Duchardt, M. Erdmann, S. Erdweg, T. Esch, R. Fischer, S. Ghosh, A. Güth, T. Hebbeker, C. Heidemann, K. Hoepfner, H. Keller, L. Mastrolorenzo, M. Merschmeyer, A. Meyer, P. Millet, S. Mukherjee, T. Pook, M. Radziej, H. Reithler, M. Rieger, A. Schmidt, D. Teyssier, S. Thüer, G. Flügge, O. Hlushchenko, T. Kress, T. Müller, A. Nehrkorn, A. Nowack, C. Pistone, O. Pooth, D. Roy, H. Sert, A. Stahl, M. Aldaya Martin, T. Arndt, C. Asawatangtrakuldee, I. Babounikau, K. Beernaert, O. Behnke, U. Behrens, A. Bermúdez Martínez, D. Bertsche, A. A. Bin Anuar, K. Borras, V. Botta, A. Campbell, P. Connor, C. Contreras-Campana, V. Danilov, A. De Wit, M. M. Defranchis, C. Diez Pardos, D. Domínguez Damiani, G. Eckerlin, T. Eichhorn, A. Elwood, E. Eren, E. Gallo, A. Geiser, J. M. Grados Luyando, A. Grohsjean, M. Guthoff, M. Haranko, A. Harb, H. Jung, M. Kasemann, J. Keaveney, C. Kleinwort, J. Knolle, D. Krücker, W. Lange, T. Lenz, J. Leonard, K. Lipka, W. Lohmann, R. Mankel, I.-A. Melzer-Pellmann, A. B. Meyer, M. Meyer, M. Missiroli, G. Mittag, J. Mnich, V. Myronenko, S. K. Pflitsch, D. Pitzl, A. Raspereza, M. Savitskyi, P. Saxena, P. Schütze, C. Schwanenberger, R. Shevchenko, A. Singh, H. Tholen, O. Turkot, A. Vagnerini, M. Van De Klundert, G. P. Van Onsem, R. Walsh, Y. Wen, K. Wichmann, C. Wissing, O. Zenaiev, R. Aggleton, S. Bein, L. Benato, A. Benecke, T. Dreyer, A. Ebrahimi, E. Garutti, D. Gonzalez, P. Gunnellini, J. Haller, A. Hinzmann, A. Karavdina, G. Kasieczka, R. Klanner, R. Kogler, N. Kovalchuk, S. Kurz, V. Kutzner, J. Lange, D. Marconi, J. Multhaup, M. Niedziela, C. E. N. Niemeyer, D. Nowatschin, A. Perieanu, A. Reimers, O. Rieger, C. Scharf, P. Schleper, S. Schumann, J. Schwandt, J. Sonneveld, H. Stadie, G. Steinbrück, F. M. Stober, M. Stöver, B. Vormwald, I. Zoi, M. Akbiyik, C. Barth, M. Baselga, S. Baur, E. Butz, R. Caspart, T. Chwalek, F. Colombo, W. De Boer, A. Dierlamm, K. El Morabit, N. Faltermann, B. Freund, M. Giffels, M. A. Harrendorf, F. Hartmann, S. M. Heindl, U. Husemann, I. Katkov, S. Kudella, S. Mitra, M. U. Mozer, Th. Müller, M. Musich, M. Plagge, G. Quast, K. Rabbertz, M. Schröder, I. Shvetsov, H. J. Simonis, R. Ulrich, S. Wayand, M. Weber, T. Weiler, C. Wöhrmann, R. Wolf, G. Anagnostou, G. Daskalakis, T. Geralis, A. Kyriakis, D. Loukas, G. Paspalaki, A. Agapitos, G. Karathanasis, P. Kontaxakis, A. Panagiotou, I. Papavergou, N. Saoulidou, E. Tziaferi, K. Vellidis, K. Kousouris, I. Papakrivopoulos, G. Tsipolitis, I. Evangelou, C. Foudas, P. Gianneios, P. Katsoulis, P. Kokkas, S. Mallios, N. Manthos, I. Papadopoulos, E. Paradas, J. Strologas, F. A. Triantis, D. Tsitsonis, M. Bartók, M. Csanad, N. Filipovic, P. Major, M. I. Nagy, G. Pasztor, O. Surányi, G. I. Veres, G. Bencze, C. Hajdu, D. Horvath, Hunyadi, F. Sikler, T. Vámi, V. Veszpremi, G. Vesztergombi, N. Beni, S. Czellar, J. Karancsi, A. Makovec, J. Molnar, Z. Szillasi, P. Raics, Z. L. Trocsanyi, B. Ujvari, S. Choudhury, J. R. Komaragiri, P. C. Tiwari, S. Bahinipati, C. Kar, P. Mal, K. Mandal, A. Nayak, S. Roy Chowdhury, D. K. Sahoo, S. K. Swain, S. Bansal, S. B. Beri, V. Bhatnagar, S. Chauhan, R. Chawla, N. Dhingra, R. Gupta, A. Kaur, M. Kaur, S. Kaur, P. Kumari, M. Lohan, M. Meena, A. Mehta, K. Sandeep, S. Sharma, J. B. Singh, A. K. Virdi, G. Walia, A. Bhardwaj, B. C. Choudhary, R. B. Garg, M. Gola, S. Keshri, Ashok Kumar, S. Malhotra, M. Naimuddin, P. Priyanka, K. Ranjan, Aashaq Shah, R. Sharma, R. Bhardwaj, M. Bharti, R. Bhattacharya, S. Bhattacharya, U. Bhawandeep, D. Bhowmik, S. Dey, S. Dutt, S. Dutta, S. Ghosh, M. Maity, K. Mondal, S. Nandan, A. Purohit, P. K. Rout, A. Roy, G. Saha, S. Sarkar, T. Sarkar, M. Sharan, B. Singh, S. Thakur, P. K. Behera, A. Muhammad, R. Chudasama, D. Dutta, V. Jha, V. Kumar, D. K. Mishra, P. K. Netrakanti, L. M. Pant, P. Shukla, P. Suggisetti, T. Aziz, M. A. Bhat, S. Dugad, G. B. Mohanty, N. Sur, RavindraKumar Verma, S. Banerjee, S. Bhattacharya, S. Chatterjee, P. Das, M. Guchait, Sa. Jain, S. Karmakar, S. Kumar, G. Majumder, K. Mazumdar, N. Sahoo, S. Chauhan, S. Dube, V. Hegde, A. Kapoor, K. Kothekar, S. Pandey, A. Rane, A. Rastogi, S. Sharma, S. Chenarani, E. Eskandari Tadavani, S. M. Etesami, M. Khakzad, M. Mohammadi Najafabadi, M. Naseri, F. Rezaei Hosseinabadi, B. Safarzadeh, M. Zeinali, M. Felcini, M. Grunewald, M. Abbrescia, C. Calabria, A. Colaleo, D. Creanza, L. Cristella, N. De Filippis, M. De Palma, A. Di Florio, F. Errico, L. Fiore, A. Gelmi, G. Iaselli, M. Ince, S. Lezki, G. Maggi, M. Maggi, G. Miniello, S. My, S. Nuzzo, A. Pompili, G. Pugliese, R. Radogna, A. Ranieri, G. Selvaggi, A. Sharma, L. Silvestris, R. Venditti, P. Verwilligen, G. Abbiendi, C. Battilana, D. Bonacorsi, L. Borgonovi, S. Braibant-Giacomelli, R. Campanini, P. Capiluppi, A. Castro, F. R. Cavallo, S. S. Chhibra, G. Codispoti, M. Cuffiani, G. M. Dallavalle, F. Fabbri, A. Fanfani, E. Fontanesi, P. Giacomelli, C. Grandi, L. Guiducci, F. Iemmi, S. Lo Meo, S. Marcellini, G. Masetti, A. Montanari, F. L. Navarria, A. Perrotta, F. Primavera, A. M. Rossi, T. Rovelli, G. P. Siroli, N. Tosi, S. Albergo, A. Di Mattia, R. Potenza, A. Tricomi, C. Tuve, G. Barbagli, K. Chatterjee, V. Ciulli, C. Civinini, R. D’Alessandro, E. Focardi, G. Latino, P. Lenzi, M. Meschini, S. Paoletti, L. Russo, G. Sguazzoni, D. Strom, L. Viliani, L. Benussi, S. Bianco, F. Fabbri, D. Piccolo, F. Ferro, R. Mulargia, E. Robutti, S. Tosi, A. Benaglia, A. Beschi, F. Brivio, V. Ciriolo, S. Di Guida, M. E. Dinardo, S. Fiorendi, S. Gennai, A. Ghezzi, P. Govoni, M. Malberti, S. Malvezzi, D. Menasce, F. Monti, L. Moroni, M. Paganoni, D. Pedrini, S. Ragazzi, T. Tabarelli de Fatis, D. Zuolo, S. Buontempo, N. Cavallo, A. De Iorio, A. Di Crescenzo, F. Fabozzi, F. Fienga, G. Galati, A. O. M. Iorio, L. Lista, S. Meola, P. Paolucci, C. Sciacca, E. Voevodina, P. Azzi, N. Bacchetta, D. Bisello, A. Boletti, A. Bragagnolo, R. Carlin, P. Checchia, M. Dall’Osso, P. De Castro Manzano, T. Dorigo, U. Dosselli, F. Gasparini, U. Gasparini, A. Gozzelino, S. Y. Hoh, S. Lacaprara, P. Lujan, M. Margoni, A. T. Meneguzzo, J. Pazzini, M. Presilla, P. Ronchese, R. Rossin, F. Simonetto, A. Tiko, E. Torassa, M. Tosi, M. Zanetti, P. Zotto, G. Zumerle, A. Braghieri, A. Magnani, P. Montagna, S. P. Ratti, V. Re, M. Ressegotti, C. Riccardi, P. Salvini, I. Vai, P. Vitulo, M. Biasini, G. M. Bilei, C. Cecchi, D. Ciangottini, L. Fanò, P. Lariccia, R. Leonardi, E. Manoni, G. Mantovani, V. Mariani, M. Menichelli, A. Rossi, A. Santocchia, D. Spiga, K. Androsov, P. Azzurri, G. Bagliesi, L. Bianchini, T. Boccali, L. Borrello, R. Castaldi, M. A. Ciocci, R. Dell’Orso, G. Fedi, F. Fiori, L. Giannini, A. Giassi, M. T. Grippo, F. Ligabue, E. Manca, G. Mandorli, A. Messineo, F. Palla, A. Rizzi, G. Rolandi, P. Spagnolo, R. Tenchini, G. Tonelli, A. Venturi, P. G. Verdini, L. Barone, F. Cavallari, M. Cipriani, D. Del Re, E. Di Marco, M. Diemoz, S. Gelli, E. Longo, B. Marzocchi, P. Meridiani, G. Organtini, F. Pandolfi, R. Paramatti, F. Preiato, S. Rahatlou, C. Rovelli, F. Santanastasio, N. Amapane, R. Arcidiacono, S. Argiro, M. Arneodo, N. Bartosik, R. Bellan, C. Biino, A. Cappati, N. Cartiglia, F. Cenna, S. Cometti, M. Costa, R. Covarelli, N. Demaria, B. Kiani, C. Mariotti, S. Maselli, E. Migliore, V. Monaco, E. Monteil, M. Monteno, M. M. Obertino, L. Pacher, N. Pastrone, M. Pelliccioni, G. L. Pinna Angioni, A. Romero, M. Ruspa, R. Sacchi, R. Salvatico, K. Shchelina, V. Sola, A. Solano, D. Soldi, A. Staiano, S. Belforte, V. Candelise, M. Casarsa, F. Cossutti, A. Da Rold, G. Della Ricca, F. Vazzoler, A. Zanetti, D. H. Kim, G. N. Kim, M. S. Kim, J. Lee, S. Lee, S. W. Lee, C. S. Moon, Y. D. Oh, S. I. Pak, S. Sekmen, D. C. Son, Y. C. Yang, H. Kim, D. H. Moon, G. Oh, B. Francois, J. Goh, T. J. Kim, S. Cho, S. Choi, Y. Go, D. Gyun, S. Ha, B. Hong, Y. Jo, K. Lee, K. S. Lee, S. Lee, J. Lim, S. K. Park, Y. Roh, H. S. Kim, J. Almond, J. Kim, J. S. Kim, H. Lee, K. Lee, K. Nam, S. B. Oh, B. C. Radburn-Smith, S. h. Seo, U. K. Yang, H. D. Yoo, G. B. Yu, D. Jeon, H. Kim, J. H. Kim, J. S. H. Lee, I. C. Park, Y. Choi, C. Hwang, J. Lee, I. Yu, V. Dudenas, A. Juodagalvis, J. Vaitkus, Z. A. Ibrahim, M. A. B. Md Ali, F. Mohamad Idris, W. A. T. Wan Abdullah, M. N. Yusli, Z. Zolkapli, J. F. Benitez, A. Castaneda Hernandez, J. A. Murillo Quijada, H. Castilla-Valdez, E. De La LaCruz-Burelo, M. C. Duran-Osuna, I. Heredia-De La Cruz, R. Lopez-Fernandez, J. Mejia Guisao, R. I. Rabadan-Trejo, M. Ramirez-Garcia, G. Ramirez-Sanchez, R. Reyes-Almanza, A. Sanchez-Hernandez, S. Carrillo Moreno, C. Oropeza Barrera, F. Vazquez Valencia, J. Eysermans, I. Pedraza, H. A. Salazar Ibarguen, C. Uribe Estrada, A. Morelos Pineda, D. Krofcheck, S. Bheesette, P. H. Butler, A. Ahmad, M. Ahmad, M. I. Asghar, Q. Hassan, H. R. Hoorani, W. A. Khan, M. A. Shah, M. Shoaib, M. Waqas, H. Bialkowska, M. Bluj, B. Boimska, T. Frueboes, M. Górski, M. Kazana, M. Szleper, P. Traczyk, P. Zalewski, K. Bunkowski, A. Byszuk, K. Doroba, A. Kalinowski, M. Konecki, J. Krolikowski, M. Misiura, M. Olszewski, A. Pyskir, M. Walczak, M. Araujo, P. Bargassa, C. Beirão Da Cruz E Silva, A. Di Francesco, P. Faccioli, B. Galinhas, M. Gallinaro, J. Hollar, N. Leonardo, J. Seixas, G. Strong, O. Toldaiev, J. Varela, S. Afanasiev, P. Bunin, M. Gavrilenko, I. Golutvin, I. Gorbunov, A. Kamenev, V. Karjavine, A. Lanev, A. Malakhov, V. Matveev, P. Moisenz, V. Palichik, V. Perelygin, S. Shmatov, S. Shulha, N. Skatchkov, V. Smirnov, N. Voytishin, A. Zarubin, V. Golovtsov, Y. Ivanov, V. Kim, E. Kuznetsova, P. Levchenko, V. Murzin, V. Oreshkin, I. Smirnov, D. Sosnov, V. Sulimov, L. Uvarov, S. Vavilov, A. Vorobyev, Yu. Andreev, A. Dermenev, S. Gninenko, N. Golubev, A. Karneyeu, M. Kirsanov, N. Krasnikov, A. Pashenkov, A. Shabanov, D. Tlisov, A. Toropin, V. Epshteyn, V. Gavrilov, N. Lychkovskaya, V. Popov, I. Pozdnyakov, G. Safronov, A. Spiridonov, A. Stepennov, V. Stolin, M. Toms, E. Vlasov, A. Zhokin, T. Aushev, M. Chadeeva, P. Parygin, E. Popova, V. Rusinov, V. Andreev, M. Azarkin, I. Dremin, M. Kirakosyan, A. Terkulov, A. Belyaev, E. Boos, M. Dubinin, L. Dudko, A. Ershov, A. Gribushin, V. Klyukhin, O. Kodolova, I. Lokhtin, S. Obraztsov, S. Petrushanko, V. Savrin, A. Snigirev, A. Barnyakov, V. Blinov, T. Dimova, L. Kardapoltsev, Y. Skovpen, I. Azhgirey, I. Bayshev, S. Bitioukov, V. Kachanov, A. Kalinin, D. Konstantinov, P. Mandrik, V. Petrov, R. Ryutin, S. Slabospitskii, A. Sobol, S. Troshin, N. Tyurin, A. Uzunian, A. Volkov, A. Babaev, S. Baidali, V. Okhotnikov, P. Adzic, P. Cirkovic, D. Devetak, M. Dordevic, J. Milosevic, J. Alcaraz Maestre, A. lvarez Fernández, I. Bachiller, M. Barrio Luna, J. A. Brochero Cifuentes, M. Cerrada, N. Colino, B. De La Cruz, A. Delgado Peris, C. Fernandez Bedoya, J. P. Fernández Ramos, J. Flix, M. C. Fouz, O. Gonzalez Lopez, S. Goy Lopez, J. M. Hernandez, M. I. Josa, D. Moran, A. Pérez-Calero Yzquierdo, J. Puerta Pelayo, I. Redondo, L. Romero, S. Sánchez Navas, M. S. Soares, A. Triossi, C. Albajar, J. F. de Trocóniz, J. Cuevas, C. Erice, J. Fernandez Menendez, S. Folgueras, I. Gonzalez Caballero, J. R. González Fernández, E. Palencia Cortezon, V. Rodríguez Bouza, S. Sanchez Cruz, J. M. Vizan Garcia, I. J. Cabrillo, A. Calderon, B. Chazin Quero, J. Duarte Campderros, M. Fernandez, P. J. Fernández Manteca, A. García Alonso, J. Garcia-Ferrero, G. Gomez, A. Lopez Virto, J. Marco, C. Martinez Rivero, P. Martinez Ruiz del Arbol, F. Matorras, J. Piedra Gomez, C. Prieels, T. Rodrigo, A. Ruiz-Jimeno, L. Scodellaro, N. Trevisani, I. Vila, R. Vilar Cortabitarte, N. Wickramage, D. Abbaneo, B. Akgun, E. Auffray, G. Auzinger, P. Baillon, A. H. Ball, D. Barney, J. Bendavid, M. Bianco, A. Bocci, C. Botta, E. Brondolin, T. Camporesi, M. Cepeda, G. Cerminara, E. Chapon, Y. Chen, G. Cucciati, D. d’Enterria, A. Dabrowski, N. Daci, V. Daponte, A. David, A. De Roeck, N. Deelen, M. Dobson, M. Dünser, N. Dupont, A. Elliott-Peisert, P. Everaerts, F. Fallavollita, D. Fasanella, G. Franzoni, J. Fulcher, W. Funk, D. Gigi, A. Gilbert, K. Gill, F. Glege, M. Gruchala, M. Guilbaud, D. Gulhan, J. Hegeman, C. Heidegger, V. Innocente, A. Jafari, P. Janot, O. Karacheban, J. Kieseler, A. Kornmayer, M. Krammer, C. Lange, P. Lecoq, C. Lourenço, L. Malgeri, M. Mannelli, A. Massironi, F. Meijers, J. A. Merlin, S. Mersi, E. Meschi, P. Milenovic, F. Moortgat, M. Mulders, J. Ngadiuba, S. Nourbakhsh, S. Orfanelli, L. Orsini, F. Pantaleo, L. Pape, E. Perez, M. Peruzzi, A. Petrilli, G. Petrucciani, A. Pfeiffer, M. Pierini, F. M. Pitters, D. Rabady, A. Racz, T. Reis, M. Rovere, H. Sakulin, C. Schäfer, C. Schwick, M. Selvaggi, A. Sharma, P. Silva, P. Sphicas, A. Stakia, J. Steggemann, D. Treille, A. Tsirou, A. Vartak, V. Veckalns, M. Verzetti, W. D. Zeuner, L. Caminada, K. Deiters, W. Erdmann, R. Horisberger, Q. Ingram, H. C. Kaestli, D. Kotlinski, U. Langenegger, T. Rohe, S. A. Wiederkehr, M. Backhaus, L. Bäni, P. Berger, N. Chernyavskaya, G. Dissertori, M. Dittmar, M. Donegà, C. Dorfer, T. A. Gómez Espinosa, C. Grab, D. Hits, T. Klijnsma, W. Lustermann, R. A. Manzoni, M. Marionneau, M. T. Meinhard, F. Micheli, P. Musella, F. Nessi-Tedaldi, F. Pauss, G. Perrin, L. Perrozzi, S. Pigazzini, C. Reissel, D. Ruini, D. A. Sanz Becerra, M. Schönenberger, L. Shchutska, V. R. Tavolaro, K. Theofilatos, M. L. Vesterbacka Olsson, R. Wallny, D. H. Zhu, T. K. Aarrestad, C. Amsler, D. Brzhechko, M. F. Canelli, A. De Cosa, R. Del Burgo, S. Donato, C. Galloni, T. Hreus, B. Kilminster, S. Leontsinis, I. Neutelings, G. Rauco, P. Robmann, D. Salerno, K. Schweiger, C. Seitz, Y. Takahashi, A. Zucchetta, T. H. Doan, R. Khurana, C. M. Kuo, W. Lin, A. Pozdnyakov, S. S. Yu, P. Chang, Y. Chao, K. F. Chen, P. H. Chen, W.-S. Hou, Y. F. Liu, R.-S. Lu, E. Paganis, A. Psallidas, A. Steen, B. Asavapibhop, N. Srimanobhas, N. Suwonjandee, A. Bat, F. Boran, S. Cerci, S. Damarseckin, Z. S. Demiroglu, F. Dolek, C. Dozen, I. Dumanoglu, E. Eskut, G. Gokbulut, Y. Guler, E. Gurpinar, I. Hos, C. Isik, E. E. Kangal, O. Kara, A. Kayis Topaksu, U. Kiminsu, M. Oglakci, G. Onengut, K. Ozdemir, A. Polatoz, B. Tali, U. G. Tok, S. Turkcapar, I. S. Zorbakir, C. Zorbilmez, B. Isildak, G. Karapinar, M. Yalvac, M. Zeyrek, I. O. Atakisi, E. Gülmez, M. Kaya, O. Kaya, S. Ozkorucuklu, S. Tekten, E. A. Yetkin, M. N. Agaras, A. Cakir, K. Cankocak, Y. Komurcu, S. Sen, B. Grynyov, L. Levchuk, F. Ball, J. J. Brooke, D. Burns, E. Clement, D. Cussans, O. Davignon, H. Flacher, J. Goldstein, G. P. Heath, H. F. Heath, L. Kreczko, D. M. Newbold, S. Paramesvaran, B. Penning, T. Sakuma, D. Smith, V. J. Smith, J. Taylor, A. Titterton, K. W. Bell, A. Belyaev, C. Brew, R. M. Brown, D. Cieri, D. J. A. Cockerill, J. A. Coughlan, K. Harder, S. Harper, J. Linacre, K. Manolopoulos, E. Olaiya, D. Petyt, C. H. Shepherd-Themistocleous, A. Thea, I. R. Tomalin, T. Williams, W. J. Womersley, R. Bainbridge, P. Bloch, J. Borg, S. Breeze, O. Buchmuller, A. Bundock, D. Colling, P. Dauncey, G. Davies, M. Della Negra, R. Di Maria, G. Hall, G. Iles, T. James, M. Komm, C. Laner, L. Lyons, A.-M. Magnan, S. Malik, A. Martelli, J. Nash, A. Nikitenko, V. Palladino, M. Pesaresi, D. M. Raymond, A. Richards, A. Rose, E. Scott, C. Seez, A. Shtipliyski, G. Singh, M. Stoye, T. Strebler, S. Summers, A. Tapper, K. Uchida, T. Virdee, N. Wardle, D. Winterbottom, J. Wright, S. C. Zenz, J. E. Cole, P. R. Hobson, A. Khan, P. Kyberd, C. K. Mackay, A. Morton, I. D. Reid, L. Teodorescu, S. Zahid, K. Call, J. Dittmann, K. Hatakeyama, H. Liu, C. Madrid, B. McMaster, N. Pastika, C. Smith, R. Bartek, A. Dominguez, A. Buccilli, S. I. Cooper, C. Henderson, P. Rumerio, C. West, D. Arcaro, T. Bose, D. Gastler, S. Girgis, D. Pinna, C. Richardson, J. Rohlf, L. Sulak, D. Zou, G. Benelli, B. Burkle, X. Coubez, D. Cutts, M. Hadley, J. Hakala, U. Heintz, J. M. Hogan, K. H. M. Kwok, E. Laird, G. Landsberg, J. Lee, Z. Mao, M. Narain, S. Sagir, R. Syarif, E. Usai, D. Yu, R. Band, C. Brainerd, R. Breedon, D. Burns, M. Calderon De La Barca Sanchez, M. Chertok, J. Conway, R. Conway, P. T. Cox, R. Erbacher, C. Flores, G. Funk, W. Ko, O. Kukral, R. Lander, M. Mulhearn, D. Pellett, J. Pilot, S. Shalhout, M. Shi, D. Stolp, D. Taylor, K. Tos, M. Tripathi, Z. Wang, F. Zhang, M. Bachtis, C. Bravo, R. Cousins, A. Dasgupta, S. Erhan, A. Florent, J. Hauser, M. Ignatenko, N. Mccoll, S. Regnard, D. Saltzberg, C. Schnaible, V. Valuev, E. Bouvier, K. Burt, R. Clare, J. W. Gary, S. M. A. Ghiasi Shirazi, G. Hanson, G. Karapostoli, E. Kennedy, F. Lacroix, O. R. Long, M. Olmedo Negrete, M. I. Paneva, W. Si, L. Wang, H. Wei, S. Wimpenny, B. R. Yates, J. G. Branson, P. Chang, S. Cittolin, M. Derdzinski, R. Gerosa, D. Gilbert, B. Hashemi, A. Holzner, D. Klein, G. Kole, V. Krutelyov, J. Letts, M. Masciovecchio, S. May, D. Olivito, S. Padhi, M. Pieri, V. Sharma, M. Tadel, J. Wood, F. Würthwein, A. Yagil, G. Zevi Della Porta, N. Amin, R. Bhandari, C. Campagnari, M. Citron, V. Dutta, M. Franco Sevilla, L. Gouskos, R. Heller, J. Incandela, H. Mei, A. Ovcharova, H. Qu, J. Richman, D. Stuart, I. Suarez, S. Wang, J. Yoo, D. Anderson, A. Bornheim, J. M. Lawhorn, N. Lu, H. B. Newman, T. Q. Nguyen, J. Pata, M. Spiropulu, J. R. Vlimant, R. Wilkinson, S. Xie, Z. Zhang, R. Y. Zhu, M. B. Andrews, T. Ferguson, T. Mudholkar, M. Paulini, M. Sun, I. Vorobiev, M. Weinberg, J. P. Cumalat, W. T. Ford, F. Jensen, A. Johnson, E. MacDonald, T. Mulholland, R. Patel, A. Perloff, K. Stenson, K. A. Ulmer, S. R. Wagner, J. Alexander, J. Chaves, Y. Cheng, J. Chu, A. Datta, K. Mcdermott, N. Mirman, J. R. Patterson, D. Quach, A. Rinkevicius, A. Ryd, L. Skinnari, L. Soffi, S. M. Tan, Z. Tao, J. Thom, J. Tucker, P. Wittich, M. Zientek, S. Abdullin, M. Albrow, M. Alyari, G. Apollinari, A. Apresyan, A. Apyan, S. Banerjee, L. A. T. Bauerdick, A. Beretvas, J. Berryhill, P. C. Bhat, K. Burkett, J. N. Butler, A. Canepa, G. B. Cerati, H. W. K. Cheung, F. Chlebana, M. Cremonesi, J. Duarte, V. D. Elvira, J. Freeman, Z. Gecse, E. Gottschalk, L. Gray, D. Green, S. Grünendahl, O. Gutsche, J. Hanlon, R. M. Harris, S. Hasegawa, J. Hirschauer, Z. Hu, B. Jayatilaka, S. Jindariani, M. Johnson, U. Joshi, B. Klima, M. J. Kortelainen, B. Kreis, S. Lammel, D. Lincoln, R. Lipton, M. Liu, T. Liu, J. Lykken, K. Maeshima, J. M. Marraffino, D. Mason, P. McBride, P. Merkel, S. Mrenna, S. Nahn, V. O’Dell, K. Pedro, C. Pena, O. Prokofyev, G. Rakness, F. Ravera, A. Reinsvold, L. Ristori, A. Savoy-Navarro, B. Schneider, E. Sexton-Kennedy, A. Soha, W. J. Spalding, L. Spiegel, S. Stoynev, J. Strait, N. Strobbe, L. Taylor, S. Tkaczyk, N. V. Tran, L. Uplegger, E. W. Vaandering, C. Vernieri, M. Verzocchi, R. Vidal, M. Wang, H. A. Weber, A. Whitbeck, D. Acosta, P. Avery, P. Bortignon, D. Bourilkov, A. Brinkerhoff, L. Cadamuro, A. Carnes, D. Curry, R. D. Field, S. V. Gleyzer, B. M. Joshi, J. Konigsberg, A. Korytov, K. H. Lo, P. Ma, K. Matchev, G. Mitselmakher, D. Rosenzweig, K. Shi, D. Sperka, J. Wang, S. Wang, X. Zuo, Y. R. Joshi, S. Linn, A. Ackert, T. Adams, A. Askew, S. Hagopian, V. Hagopian, K. F. Johnson, T. Kolberg, G. Martinez, T. Perry, H. Prosper, A. Saha, C. Schiber, R. Yohay, M. M. Baarmand, V. Bhopatkar, S. Colafranceschi, M. Hohlmann, D. Noonan, M. Rahmani, T. Roy, M. Saunders, F. Yumiceva, M. R. Adams, L. Apanasevich, D. Berry, R. R. Betts, R. Cavanaugh, X. Chen, S. Dittmer, O. Evdokimov, C. E. Gerber, D. A. Hangal, D. J. Hofman, K. Jung, J. Kamin, C. Mills, M. B. Tonjes, N. Varelas, H. Wang, X. Wang, Z. Wu, J. Zhang, M. Alhusseini, B. Bilki, W. Clarida, K. Dilsiz, S. Durgut, R. P. Gandrajula, M. Haytmyradov, V. Khristenko, J.-P. Merlo, A. Mestvirishvili, A. Moeller, J. Nachtman, H. Ogul, Y. Onel, F. Ozok, A. Penzo, C. Snyder, E. Tiras, J. Wetzel, B. Blumenfeld, A. Cocoros, N. Eminizer, D. Fehling, L. Feng, A. V. Gritsan, W. T. Hung, P. Maksimovic, J. Roskes, U. Sarica, M. Swartz, M. Xiao, A. Al-bataineh, P. Baringer, A. Bean, S. Boren, J. Bowen, A. Bylinkin, J. Castle, S. Khalil, A. Kropivnitskaya, D. Majumder, W. Mcbrayer, M. Murray, C. Rogan, S. Sanders, E. Schmitz, J. D. Tapia Takaki, Q. Wang, S. Duric, A. Ivanov, K. Kaadze, D. Kim, Y. Maravin, D. R. Mendis, T. Mitchell, A. Modak, A. Mohammadi, F. Rebassoo, D. Wright, A. Baden, O. Baron, A. Belloni, S. C. Eno, Y. Feng, C. Ferraioli, N. J. Hadley, S. Jabeen, G. Y. Jeng, R. G. Kellogg, J. Kunkle, A. C. Mignerey, S. Nabili, F. Ricci-Tam, M. Seidel, Y. H. Shin, A. Skuja, S. C. Tonwar, K. Wong, D. Abercrombie, B. Allen, V. Azzolini, A. Baty, R. Bi, S. Brandt, W. Busza, I. A. Cali, M. D’Alfonso, Z. Demiragli, G. Gomez Ceballos, M. Goncharov, P. Harris, D. Hsu, M. Hu, Y. Iiyama, G. M. Innocenti, M. Klute, D. Kovalskyi, Y.-J. Lee, P. D. Luckey, B. Maier, A. C. Marini, C. Mcginn, C. Mironov, S. Narayanan, X. Niu, C. Paus, D. Rankin, C. Roland, G. Roland, Z. Shi, G. S. F. Stephans, K. Sumorok, K. Tatar, D. Velicanu, J. Wang, T. W. Wang, B. Wyslouch, A. C. Benvenuti, R. M. Chatterjee, A. Evans, P. Hansen, J. Hiltbrand, Sh. Jain, S. Kalafut, M. Krohn, Y. Kubota, Z. Lesko, J. Mans, R. Rusack, M. A. Wadud, J. G. Acosta, S. Oliveros, E. Avdeeva, K. Bloom, D. R. Claes, C. Fangmeier, F. Golf, R. Gonzalez Suarez, R. Kamalieddin, I. Kravchenko, J. Monroy, J. E. Siado, G. R. Snow, B. Stieger, A. Godshalk, C. Harrington, I. Iashvili, A. Kharchilava, C. Mclean, D. Nguyen, A. Parker, S. Rappoccio, B. Roozbahani, G. Alverson, E. Barberis, C. Freer, Y. Haddad, A. Hortiangtham, G. Madigan, D. M. Morse, T. Orimoto, A. Tishelman-charny, T. Wamorkar, B. Wang, A. Wisecarver, D. Wood, S. Bhattacharya, J. Bueghly, O. Charaf, T. Gunter, K. A. Hahn, N. Odell, M. H. Schmitt, K. Sung, M. Trovato, M. Velasco, R. Bucci, N. Dev, M. Hildreth, K. Hurtado Anampa, C. Jessop, D. J. Karmgard, K. Lannon, W. Li, N. Loukas, N. Marinelli, F. Meng, C. Mueller, Y. Musienko, M. Planer, R. Ruchti, P. Siddireddy, G. Smith, S. Taroni, M. Wayne, A. Wightman, M. Wolf, A. Woodard, J. Alimena, L. Antonelli, B. Bylsma, L. S. Durkin, S. Flowers, B. Francis, C. Hill, W. Ji, T. Y. Ling, W. Luo, B. L. Winer, S. Cooperstein, P. Elmer, J. Hardenbrook, N. Haubrich, S. Higginbotham, A. Kalogeropoulos, S. Kwan, D. Lange, M. T. Lucchini, J. Luo, D. Marlow, K. Mei, I. Ojalvo, J. Olsen, C. Palmer, P. Piroué, J. Salfeld-Nebgen, D. Stickland, C. Tully, S. Malik, S. Norberg, A. Barker, V. E. Barnes, S. Das, L. Gutay, M. Jones, A. W. Jung, A. Khatiwada, B. Mahakud, D. H. Miller, N. Neumeister, C. C. Peng, S. Piperov, H. Qiu, J. F. Schulte, J. Sun, F. Wang, R. Xiao, W. Xie, T. Cheng, J. Dolen, N. Parashar, Z. Chen, K. M. Ecklund, S. Freed, F. J. M. Geurts, M. Kilpatrick, Arun Kumar, W. Li, B. P. Padley, R. Redjimi, J. Roberts, J. Rorie, W. Shi, Z. Tu, A. Zhang, A. Bodek, P. de Barbaro, R. Demina, Y. T. Duh, J. L. Dulemba, C. Fallon, T. Ferbel, M. Galanti, A. Garcia-Bellido, J. Han, O. Hindrichs, A. Khukhunaishvili, E. Ranken, P. Tan, R. Taus, B. Chiarito, J. P. Chou, Y. Gershtein, E. Halkiadakis, A. Hart, M. Heindl, E. Hughes, S. Kaplan, R. Kunnawalkam Elayavalli, S. Kyriacou, I. Laflotte, A. Lath, R. Montalvo, K. Nash, M. Osherson, H. Saka, S. Salur, S. Schnetzer, D. Sheffield, S. Somalwar, R. Stone, S. Thomas, P. Thomassen, A. G. Delannoy, J. Heideman, G. Riley, S. Spanier, O. Bouhali, A. Celik, M. Dalchenko, M. De Mattia, A. Delgado, S. Dildick, R. Eusebi, J. Gilmore, T. Huang, T. Kamon, S. Luo, D. Marley, R. Mueller, D. Overton, L. Perniè, D. Rathjens, A. Safonov, N. Akchurin, J. Damgov, F. De Guio, P. R. Dudero, S. Kunori, K. Lamichhane, S. W. Lee, T. Mengke, S. Muthumuni, T. Peltola, S. Undleeb, I. Volobouev, Z. Wang, S. Greene, A. Gurrola, R. Janjam, W. Johns, C. Maguire, A. Melo, H. Ni, K. Padeken, F. Romeo, J. D. Ruiz Alvarez, P. Sheldon, S. Tuo, J. Velkovska, M. Verweij, Q. Xu, M. W. Arenton, P. Barria, B. Cox, R. Hirosky, M. Joyce, A. Ledovskoy, H. Li, C. Neu, T. Sinthuprasith, Y. Wang, E. Wolfe, F. Xia, R. Harr, P. E. Karchin, N. Poudyal, J. Sturdy, P. Thapa, S. Zaleski, J. Buchanan, C. Caillol, D. Carlsmith, S. Dasu, I. De Bruyn, L. Dodd, B. Gomber, M. Grothe, M. Herndon, A. Hervé, U. Hussain, P. Klabbers, A. Lanaro, K. Long, R. Loveless, T. Ruggles, A. Savin, V. Sharma, N. Smith, W. H. Smith, N. Woods, CMS Collaboration

**Affiliations:** 1 Yerevan Physics Institute, Yerevan, Armenia; 2 Institut für Hochenergiephysik, Wien, Austria; 3 Institute for Nuclear Problems, Minsk, Belarus; 4 Universiteit Antwerpen, Antwerpen, Belgium; 5 Vrije Universiteit Brussel, Brussel, Belgium; 6 Université Libre de Bruxelles, Bruxelles, Belgium; 7 Ghent University, Ghent, Belgium; 8 Université Catholique de Louvain, Louvain-la-Neuve, Belgium; 9 Centro Brasileiro de Pesquisas Fisicas, Rio de Janeiro, Brazil; 10 Universidade do Estado do Rio de Janeiro, Rio de Janeiro, Brazil; 11 Universidade Estadual Paulista, Universidade Federal do ABC, São Paulo, Brazil; 12 Institute for Nuclear Research and Nuclear Energy, Bulgarian Academy of Sciences, Sofia, Bulgaria; 13 University of Sofia, Sofia, Bulgaria; 14 Beihang University, Beijing, China; 15 Institute of High Energy Physics, Beijing, China; 16 State Key Laboratory of Nuclear Physics and Technology, Peking University, Beijing, China; 17 Tsinghua University, Beijing, China; 18 Universidad de Los Andes, Bogota, Colombia; 19 University of Split, Faculty of Electrical Engineering, Mechanical Engineering and Naval Architecture, Split, Croatia; 20 University of Split, Faculty of Science, Split, Croatia; 21 Institute Rudjer Boskovic, Zagreb, Croatia; 22 University of Cyprus, Nicosia, Cyprus; 23 Charles University, Prague, Czech Republic; 24 Escuela Politecnica Nacional, Quito, Ecuador; 25 Universidad San Francisco de Quito, Quito, Ecuador; 26 Academy of Scientific Research and Technology of the Arab Republic of Egypt, Egyptian Network of High Energy Physics, Cairo, Egypt; 27 National Institute of Chemical Physics and Biophysics, Tallinn, Estonia; 28 Department of Physics, University of Helsinki, Helsinki, Finland; 29 Helsinki Institute of Physics, Helsinki, Finland; 30 Lappeenranta University of Technology, Lappeenranta, Finland; 31 IRFU, CEA, Université Paris-Saclay, Gif-sur-Yvette, France; 32 Laboratoire Leprince-Ringuet, Ecole polytechnique, CNRS/IN2P3, Université Paris-Saclay, Palaiseau, France; 33 Université de Strasbourg, CNRS, IPHC UMR 7178, Strasbourg, France; 34 Centre de Calcul de l’Institut National de Physique Nucleaire et de Physique des Particules, CNRS/IN2P3, Villeurbanne, France; 35 Université de Lyon, Université Claude Bernard Lyon 1, CNRS-IN2P3, Institut de Physique Nucléaire de Lyon, Villeurbanne, France; 36 Georgian Technical University, Tbilisi, Georgia; 37 Tbilisi State University, Tbilisi, Georgia; 38 RWTH Aachen University, I. Physikalisches Institut, Aachen, Germany; 39 RWTH Aachen University, III. Physikalisches Institut A, Aachen, Germany; 40 RWTH Aachen University, III. Physikalisches Institut B, Aachen, Germany; 41 Deutsches Elektronen-Synchrotron, Hamburg, Germany; 42 University of Hamburg, Hamburg, Germany; 43 Karlsruher Institut fuer Technologie, Karlsruhe, Germany; 44 Institute of Nuclear and Particle Physics (INPP), NCSR Demokritos, Aghia Paraskevi, Greece; 45 National and Kapodistrian University of Athens, Athens, Greece; 46 National Technical University of Athens, Athens, Greece; 47 University of Ioánnina, Ioánnina, Greece; 48 MTA-ELTE Lendület CMS Particle and Nuclear Physics Group, Eötvös Loránd University, Budapest, Hungary; 49 Wigner Research Centre for Physics, Budapest, Hungary; 50 Institute of Nuclear Research ATOMKI, Debrecen, Hungary; 51 Institute of Physics, University of Debrecen, Debrecen, Hungary; 52 Indian Institute of Science (IISc), Bangalore, India; 53 National Institute of Science Education and Research, HBNI, Bhubaneswar, India; 54 Panjab University, Chandigarh, India; 55 University of Delhi, Delhi, India; 56 Saha Institute of Nuclear Physics, HBNI, Kolkata, India; 57 Indian Institute of Technology Madras, Madras, India; 58 Bhabha Atomic Research Centre, Mumbai, India; 59 Tata Institute of Fundamental Research-A, Mumbai, India; 60 Tata Institute of Fundamental Research-B, Mumbai, India; 61 Indian Institute of Science Education and Research (IISER), Pune, India; 62 Institute for Research in Fundamental Sciences (IPM), Tehran, Iran; 63 University College Dublin, Dublin, Ireland; 64 INFN Sezione di Bari, Università di Bari, Politecnico di Bari, Bari, Italy; 65 INFN Sezione di Bologna, Università di Bologna, Bologna, Italy; 66 INFN Sezione di Catania, Università di Catania, Catania, Italy; 67 INFN Sezione di Firenze, Università di Firenze, Firenze, Italy; 68 INFN Laboratori Nazionali di Frascati, Frascati, Italy; 69 INFN Sezione di Genova, Università di Genova, Genova, Italy; 70 INFN Sezione di Milano-Bicocca, Università di Milano-Bicocca, Milano, Italy; 71 INFN Sezione di Napoli, Università di Napoli ’Federico II’ , Napoli, Italy, Università della Basilicata, Potenza, Italy, Università G. Marconi, Roma, Italy; 72 INFN Sezione di Padova, Università di Padova, Padova, Italy, Università di Trento, Trento, Italy; 73 INFN Sezione di Pavia, Università di Pavia, Pavia, Italy; 74 INFN Sezione di Perugia, Università di Perugia, Perugia, Italy; 75 INFN Sezione di Pisa, Università di Pisa, Scuola Normale Superiore di Pisa, Pisa, Italy; 76 INFN Sezione di Roma, Sapienza Università di Roma, Rome, Italy; 77 INFN Sezione di Torino, Università di Torino, Torino, Italy, Università del Piemonte Orientale, Novara, Italy; 78 INFN Sezione di Trieste, Università di Trieste, Trieste, Italy; 79 Kyungpook National University, Daegu, Korea; 80 Chonnam National University, Institute for Universe and Elementary Particles, Kwangju, Korea; 81 Hanyang University, Seoul, Korea; 82 Korea University, Seoul, Korea; 83 Sejong University, Seoul, Korea; 84 Seoul National University, Seoul, Korea; 85 University of Seoul, Seoul, Korea; 86 Sungkyunkwan University, Suwon, Korea; 87 Vilnius University, Vilnius, Lithuania; 88 National Centre for Particle Physics, Universiti Malaya, Kuala Lumpur, Malaysia; 89 Universidad de Sonora (UNISON), Hermosillo, Mexico; 90 Centro de Investigacion y de Estudios Avanzados del IPN, Mexico City, Mexico; 91 Universidad Iberoamericana, Mexico City, Mexico; 92 Benemerita Universidad Autonoma de Puebla, Puebla, Mexico; 93 Universidad Autónoma de San Luis Potosí, San Luis Potosí, Mexico; 94 University of Auckland, Auckland, New Zealand; 95 University of Canterbury, Christchurch, New Zealand; 96 National Centre for Physics, Quaid-I-Azam University, Islamabad, Pakistan; 97 National Centre for Nuclear Research, Swierk, Poland; 98 Institute of Experimental Physics, Faculty of Physics, University of Warsaw, Warsaw, Poland; 99 Laboratório de Instrumentação e Física Experimental de Partículas, Lisboa, Portugal; 100 Joint Institute for Nuclear Research, Dubna, Russia; 101 Petersburg Nuclear Physics Institute, Gatchina (St. Petersburg), Russia; 102 Institute for Nuclear Research, Moscow, Russia; 103 Institute for Theoretical and Experimental Physics named by A.I. Alikhanov of NRC ‘Kurchatov Institute’, Moscow, Russia; 104 Moscow Institute of Physics and Technology, Moscow, Russia; 105 National Research Nuclear University ’Moscow Engineering Physics Institute’ (MEPhI), Moscow, Russia; 106 P.N. Lebedev Physical Institute, Moscow, Russia; 107 Skobeltsyn Institute of Nuclear Physics, Lomonosov Moscow State University, Moscow, Russia; 108 Novosibirsk State University (NSU), Novosibirsk, Russia; 109 Institute for High Energy Physics of National Research Centre ‘Kurchatov Institute’, Protvino, Russia; 110 National Research Tomsk Polytechnic University, Tomsk, Russia; 111 University of Belgrade: Faculty of Physics and VINCA Institute of Nuclear Sciences, Belgrade , Serbia; 112 Centro de Investigaciones Energéticas Medioambientales y Tecnológicas (CIEMAT), Madrid, Spain; 113 Universidad Autónoma de Madrid, Madrid, Spain; 114 Universidad de Oviedo, Instituto Universitario de Ciencias y Tecnologías Espaciales de Asturias (ICTEA), Oviedo , Spain; 115 Instituto de Física de Cantabria (IFCA), CSIC-Universidad de Cantabria, Santander, Spain; 116 Department of Physics, University of Ruhuna, Matara, Sri Lanka; 117 CERN, European Organization for Nuclear Research, Geneva, Switzerland; 118 Paul Scherrer Institut, Villigen, Switzerland; 119 ETH Zurich - Institute for Particle Physics and Astrophysics (IPA), Zurich, Switzerland; 120 Universität Zürich, Zurich, Switzerland; 121 National Central University, Chung-Li, Taiwan; 122 National Taiwan University (NTU), Taipei, Taiwan; 123 Chulalongkorn University, Faculty of Science, Department of Physics, Bangkok, Thailand; 124 ukurova University, Physics Department, Science and Art Faculty, Adana, Turkey; 125 Middle East Technical University, Physics Department, Ankara, Turkey; 126 Bogazici University, Istanbul, Turkey; 127 Istanbul Technical University, Istanbul, Turkey; 128 Institute for Scintillation Materials of National Academy of Science of Ukraine, Kharkov, Ukraine; 129 National Scientific Center, Kharkov Institute of Physics and Technology, Kharkov, Ukraine; 130 University of Bristol, Bristol, United Kingdom; 131 Rutherford Appleton Laboratory, Didcot, United Kingdom; 132 Imperial College, London, United Kingdom; 133 Brunel University, Uxbridge, United Kingdom; 134 Baylor University, Waco, USA; 135 Catholic University of America, Washington DC, USA; 136 The University of Alabama, Tuscaloosa, USA; 137 Boston University, Boston, USA; 138 Brown University, Providence, USA; 139 University of California, Davis, Davis USA; 140 University of California, Los Angeles, USA; 141 University of California, Riverside, Riverside, USA; 142 University of California, San Diego, La Jolla, USA; 143 University of California, Santa Barbara-Department of Physics, Santa Barbara, USA; 144 California Institute of Technology, Pasadena, USA; 145 Carnegie Mellon University, Pittsburgh, USA; 146 University of Colorado Boulder, Boulder, USA; 147 Cornell University, Ithaca, USA; 148 Fermi National Accelerator Laboratory, Batavia, USA; 149 University of Florida, Gainesville, USA; 150 Florida International University, Miami, USA; 151 Florida State University, Tallahassee, USA; 152 Florida Institute of Technology, Melbourne, USA; 153 University of Illinois at Chicago (UIC), Chicago, USA; 154 The University of Iowa, Iowa City, USA; 155 Johns Hopkins University, Baltimore, USA; 156 The University of Kansas, Lawrence, USA; 157 Kansas State University, Manhattan, USA; 158 Lawrence Livermore National Laboratory, Livermore, USA; 159 University of Maryland, College Park, USA; 160 Massachusetts Institute of Technology, Cambridge, USA; 161 University of Minnesota, Minneapolis, USA; 162 University of Mississippi, Oxford, USA; 163 University of Nebraska-Lincoln, Lincoln, USA; 164 State University of New York at Buffalo, Buffalo, USA; 165 Northeastern University, Boston, USA; 166 Northwestern University, Evanston, USA; 167 University of Notre Dame, Notre Dame, USA; 168 The Ohio State University, Columbus, USA; 169 Princeton University, Princeton, USA; 170 University of Puerto Rico, Mayaguez, USA; 171 Purdue University, West Lafayette, USA; 172 Purdue University Northwest, Hammond, USA; 173 Rice University, Houston, USA; 174 University of Rochester, Rochester, USA; 175 Rutgers, The State University of New Jersey, Piscataway, USA; 176 University of Tennessee, Knoxville, USA; 177 Texas A & M University, College Station, USA; 178 Texas Tech University, Lubbock, USA; 179 Vanderbilt University, Nashville, USA; 180 University of Virginia, Charlottesville, USA; 181 Wayne State University, Detroit, USA; 182 University of Wisconsin - Madison, Madison, WI USA; 183 CERN, 1211 Geneva 23, Switzerland

## Abstract

Measurements are presented of the triple-differential cross section for inclusive isolated-photon+jet events in $\mathrm{p}\mathrm{p}$ collisions at $\sqrt{s} = 8$ TeV as a function of photon transverse momentum ($p_{\mathrm {T}} ^{{\upgamma {}{}}}$), photon pseudorapidity ($\eta ^{{\upgamma {}{}}}$), and jet pseudorapidity ($\eta ^{\text {jet}}$). The data correspond to an integrated luminosity of $19.7{\,\text {fb}^{-1}}$ that probe a broad range of the available phase space, for $|\eta ^{{\upgamma {}{}}} |<1.44$ and $1.57<|\eta ^{{\upgamma {}{}}} |<2.50$, $|\eta ^{\text {jet}} |<2.5$, $40< p_{\mathrm {T}} ^{{\upgamma {}{}}}<1000$ $\,\text {GeV}$, and jet transverse momentum, $p_{\mathrm {T}} ^{\text {jet}}$, > 25$\,\text {GeV}$. The measurements are compared to next-to-leading order perturbative quantum chromodynamics calculations, which reproduce the data within uncertainties.

## Introduction

Direct photons produced in the hard scattering of partons in proton–proton collisions are sensitive probes of the perturbative regime of quantum chromodynamics (pQCD) [1, 2] and provide useful constraints on the parton distribution function (PDF) of gluons [3–5]. At leading order in pQCD, direct photons are produced mainly through quark-gluon scattering (${\mathrm{q}} \mathrm{g}\rightarrow {\mathrm{q}} {\upgamma {}{}}$) with smaller contributions from quark antiquark annihilation (${q}\bar{q}\rightarrow \mathrm{g}{\upgamma {}{}}$). Photons can also be produced via fragmentation of the final state partons. These latter photons are typically accompanied by other partons, and their contributions can be experimentally suppressed by requiring the photons to be isolated from other energy depositions in the calorimeters. A good understanding of isolated photon production also indirectly impacts all jet measurements at the LHC, because photon+jet events are commonly used to determine the absolute jet energy-scale. This process also constitutes a main background in important standard model (SM) processes, such as $\mathrm{H}\rightarrow {\upgamma {}{}} {\upgamma {}{}}$, as well as in searches for physics beyond the SM.

This paper presents measurements of the triple-differential inclusive isolated-photon+jet cross sections using data collected by the CMS experiment during the 2012 run at $\sqrt{s} = 8\,\text {TeV}$ corresponding to an integrated luminosity of $19.7{\,\text {fb}^{-1}}$. Measurement of the cross section as a function of different combinations of photon and jet pseudorapidities in the range of $|\eta |<2.5$ allows for the exploration of parton collisions at different values of momentum transfer squared ($Q^{2}$) and parton momentum fraction (*x*). Given the photon transverse momentum range of $p_{\mathrm {T}} ^{{\upgamma {}{}}}= 40$–1000$\,\text {GeV}$, the measurement probes $Q^{2}= (p_{\mathrm {T}} ^{{\upgamma {}{}}})^{2}$ in the range $10^{3}$–$10^{6}\,\text {GeV} ^{2}$, and $x_\text {T}={2p_{\mathrm {T}} ^{{\upgamma {}{}}}}/{\sqrt{s}}$ in the range 0.01–0.25, where $x_\text {T}$ is an approximation to the parton momentum fraction when both photon and jet are produced centrally. This measurement is complementary to previous ones [6–11] in the coverage of the $Q^{2}-x$ phase space. The cross section can be written as:

1 $$\begin{aligned} \left( \frac{{\mathrm{d}}^{3}\sigma }{{\mathrm{d}}p_{\mathrm {T}} ^{{\upgamma {}{}}}{\mathrm{d}}|\eta ^{{\upgamma {}{}}} |{\mathrm{d}}|\eta ^{\text {jet}} |}\right) _{i}= \frac{1}{{\varDelta p_{\mathrm {T}} ^{{\upgamma {}{}}}}_{i}{\varDelta |\eta ^{{\upgamma {}{}}} |}_{i}{\varDelta |\eta ^{\text {jet}} |}_{i}}{\sum }_{j} U_{ij} \frac{N_{i} p_{i}}{\epsilon _{i} {\mathcal {L} ^{'}}_{i}}, \end{aligned}$$

where $N_i$ is the number of candidate events, $p_i$ is the signal purity, $\epsilon _i$ is the detection efficiency, ${\mathcal {L} ^{'}}_i$ is the effective integrated luminosity, and ${\varDelta p_{\mathrm {T}} ^{{\upgamma {}{}}}}_i$, ${\varDelta |\eta ^{{\upgamma {}{}}} |}_i$, and ${\varDelta |\eta ^{\text {jet}} |}_i$ are the bin size in $p_{\mathrm {T}} ^{{\upgamma {}{}}}$, $|\eta ^{{\upgamma {}{}}} |$, and $|\eta ^{\text {jet}} |$ in the *i*th data bin. $U_{ij}$ is the coefficient of the unfolding matrix between the true quantity in bin *j* and measured quantities in bin *i*.

The paper is organized as follows. Section 2 provides a brief introduction to the CMS detector. Selection and reconstruction of events, with attention focused on issues of triggering, photon reconstruction, selections and efficiency, are detailed in Sect. 3. Section 4 describes the extraction of the signal photons from the energy depositions that originate from neutral meson decays, the unfolding, and the measurement of differential cross sections. The results of the measurement, along with comparison with theoretical predictions, are reported in Sect. 5. Finally, the summary is presented in Sect. 6.

## The CMS detector

A detailed description of the CMS detector, together with definitions of the coordinate system and relevant kinematic variables, is presented in Ref. [12]. The central feature of the CMS apparatus is a superconducting solenoid of $6\,\text {m}$ internal diameter, providing a magnetic field of $3.8\,\text {T}$. Within the field volume are a silicon pixel and strip tracker, a lead tungstate crystal electromagnetic calorimeter (ECAL), and a brass and plastic scintillator hadronic calorimeter (HCAL), each composed of a barrel and two endcap sections. Muons are measured in gas-ionization detectors embedded in the steel flux-return yoke outside the solenoid. Extensive forward calorimetry complements the coverage provided by the barrel and endcap detectors.

## Event reconstruction and selection

The particle-flow algorithm [13] reconstructs and identifies each individual particle with an optimized combination of information from the various elements of the CMS detector. The identification and energy measurement of muons, electrons, photons, hadronic jets as well as the missing transverse momentum come from particle-flow objects. In addition, the isolations of identified leptons and photons are measured using the $p_{\mathrm {T}}$ of particle-flow charged hadrons, photons, and neutral hadrons. Jets are reconstructed using the anti-$k_{{\mathrm{T}}}$ algorithm with a distance parameter of $\varDelta R= 0.5$ [14], where *R* determines the size of the jet in $\eta$–$\phi$ space and $\phi$ is measured in radians. Corrections are applied to the jet energy as functions of jet $\eta$ and $p_{\mathrm {T}}$ to account for contributions from additional inelastic proton-proton interactions in the same or neighboring bunch crossings (pileup), and for the nonuniform and nonlinear response of the detectors [15]. Jets are further required to have at least minimal energy depositions in the tracker, HCAL, and ECAL to reject spurious jets associated with calorimeter noise as well as those associated with muon and electron candidates that are either mis-reconstructed or isolated [16]. Jets have typical energy resolutions of 15–20% at 30$\,\text {GeV}$, 10% at 100$\,\text {GeV}$, and 5% at 1$\,\text {TeV}$ [13].

Photons are selected from clusters of energy measured in the ECAL with a small corresponding energy deposition in the HCAL. For the reconstruction of the endcap photons, the depositions of energy in the preshower detector are also included. The calorimeter signals are calibrated and corrected for changes in the detector response over time. The energy resolution of isolated photons is about 1% in the barrel section of the ECAL for unconverted photons (photons that did not convert to electrons before reaching the ECAL) in the tens of $\,\text {GeV}$ energy range. The remaining barrel photons in the similar energy range have a resolution of about 1.3% up to a pseudorapidity of $|\eta |= 1.0$, rising to about 2.5% at $|\eta |= 1.4$. In the endcaps, the resolution of unconverted photons is about 2.5%, while the remaining endcap photons have a resolution between 3 and 4% [17].

Muons are identified by tracks in the muon spectrometer matched to tracks in the silicon tracker. Quality requirements are placed on the silicon tracker and muon spectrometer track measurements as well as on the matching between them. Matching muon spectrometer tracks to tracks measured in the silicon tracker results in a relative $p_{\mathrm {T}}$ resolution of 1.3–2.0% for muons in the momentum range $20<p_{\mathrm {T}} < 100\,\text {GeV}$ in the barrel ($|\eta |<1.2$) and better than 6% in the endcaps ($1.2<|\eta |<2.4$) [18].

Events selected for this analysis are recorded using a two-level trigger system [19]. A hardware based level-1 trigger requires a cluster of energy deposited within the ECAL above a pre-defined $p_{\mathrm {T}}$ threshold. This threshold is $p_{\mathrm {T}} >20$ or 22$\,\text {GeV}$, and is raised to 30$\,\text {GeV}$ at high luminosity to keep trigger rates at manageable levels. The CMS high-level trigger (HLT) applies a more complicated ECAL energy clustering algorithm than that of level-1, and requires additional $p_{\mathrm {T}}$ trigger thresholds ranging from 30 to 150$\,\text {GeV}$. HLT triggers with thresholds below 90$\,\text {GeV}$ have additional loose calorimetric identification requirements, based on the electromagnetic (EM) shower, and are prescaled such that only a fraction of events satisfying the trigger requirements are recorded. Since the trigger rates for lower $p_{\mathrm {T}}$ threshold triggers are controlled by applying larger prescale factors, the effective luminosity is smaller for the lower $p_{\mathrm {T}}$ regions. Triggers are combined for different $p_{\mathrm {T}}$ ranges to maximize the number of events without loss of efficiency.

Samples of simulated events used for signal and background studies are described below. Events from both photon+jet production and QCD multijet production with enhanced EM content are generated using ${\textsc {pythia}}$ version 6.426 [20], and passed through the full CMS detector simulation implemented in ${\textsc {Geant4}}$ [21]. The EM-enriched QCD sample is generated by applying a filter that is designed to enhance the production efficiency of fake photons from jets with EM fluctuations. The filter accepts events having photons, electrons, or neutral hadrons with: (i) a $p_{\mathrm {T}} >15\,\text {GeV}$ within a small region, and (ii) no more than one charged particle in a cone of $\varDelta R= \sqrt{\smash [b]{(\varDelta \eta )^2+(\varDelta \phi )^2}} < 0.2$. Samples for reconstruction efficiency studies of inclusive $\mathrm{Z}/{\upgamma {}{}} ^{*} \rightarrow \mathrm{e}^+\mathrm{e}^-$ and $\mathrm{Z}/{\upgamma {}{}} ^{*} \rightarrow \upmu ^+\upmu ^-{\upgamma {}{}}$ are generated using $\textsc {MadGraph}$ 5.1.5.11 [22]. For generation purposes, the CTEQ6L [23] parton distribution functions are used along with underlying event tune Z2* [24] for all MC samples. All the samples include simulation of the multiple $\mathrm{p}$ $\mathrm{p}$ interactions taking place in each bunch crossing, which are weighted to produce the pileup distribution observed in data.

Events selected with the single-photon trigger are chosen offline by requiring at least one photon candidate with $p_{\mathrm {T}} ^{{\upgamma {}{}}}>40$ $\,\text {GeV}$. Photon candidates must either be in the barrel ($|\eta |<1.44$) or endcap ($1.57<|\eta ^{{\upgamma {}{}}} |<2.50$) detector regions. The leading jet is required to be separated from the photon candidate by $\varDelta R>0.5$, pass the jet identification requirements, and have $p_{\mathrm {T}} ^{\text {jet}}>25$ $\,\text {GeV}$  and $|\eta |<2.5$. Therefore, dijet events where a photon is radiated in a parton shower are included.

The dominant background originates from the decays of neutral hadrons, such as ${\uppi ^{0}}$ and ${\upeta {}{}}$ mesons, into photon pairs with small angular separation. To separate signal photons from this background, photons are selected by requiring a narrow transverse shower shape in the ECAL (in the $\eta$ coordinate), no matching reconstructed track candidates (except for electron tracks from photon conversion), and minimal energy measured in the HCAL region matched to the ECAL shower. Photon candidates are further required to be isolated from nearby particle-flow candidates, such as charged hadrons and photons, after removing those consistent with pileup [17]. A photon candidate is defined as isolated from charged hadrons if the sum of the $p_{\mathrm {T}}$ of the charged hadron particle-flow candidates in a cone of radius $\varDelta R<0.3$ around its direction is less than 5$\,\text {GeV}$. To limit correlations of the selected photon candidate’s shower energy with other photon quantities, an area in the vicinity of the photon candidate is eliminated in the calculation of the photon isolation (calculated similarly to charged hadron isolation but from the $p_{\mathrm {T}}$ sum of the photon particle-flow candidates), leading to smaller correlation overall. Because of the pileup subtraction, the final photon isolation may be negative as calculated. Final photon candidates are required to have less than 0.0$\,\text {GeV}$ for $|\eta |<1.44$, $-0.5\,\text {GeV}$ for $1.5<|\eta |<2.1$, and $-1.0\,\text {GeV}$ for $2.1<|\eta |<2.5$.

Several quantities related to the shape of the EM shower are then used in a boosted-decision-tree (BDT) [25] to discriminate between direct photons and photons from hadronic activity. These quantities include the transverse width of the cluster in the $\eta$ and $\phi$ coordinates in the ECAL, the calorimetry-based likelihood of this shower to come from a conversion, the pseudorapidity of the cluster, and the average pileup energy density of the event. Simulated samples of photons originating from photon+jet events, where the reconstructed photons are matched to the generated photon, are used as training samples for the signal. Samples of simulated QCD multijet events selected at generation level as containing electromagnetically decaying final particles are used for background training. The background contribution from electrons misidentified as photons is determined from simulation, using $\mathrm{W}\rightarrow \mathrm{e}\nu$ sample, and found to be many orders of magnitude smaller than the QCD multijet background. Therefore, this background is not considered in the BDT training. The output from this BDT is then used to statistically quantify the fraction of true photons in the candidate sample.

The efficiency of the photon selection is estimated from simulated photon+jet events. To validate the efficiency, large samples of $\mathrm{Z}\rightarrow \mathrm{e}^+\mathrm{e}^-$ events in data and simulation are compared. Since the electrons at CMS are reconstructed by pairing ECAL energy depositions with the tracks in the tracker, electron showers can be reconstructed as photons to validate photon selection and identification. The trigger efficiency is measured to be approximately 100 (97)% with an uncertainty of $\approx$3 (2)% for barrel (endcap) events above the corresponding trigger thresholds. To maintain well-defined trigger efficiencies and effective luminosities, the bins for the cross section are chosen so that maximum efficiency is maintained for each trigger with a separate threshold. The photon selection efficiencies for the offline preselection and isolation criteria are estimated to be $84\pm 3.4$, $83\pm 6.2$, $81\pm 6.5$, and $88\pm 10.1\%$ in $|\eta |<0.8$, $0.8<|\eta |<1.44$, $1.56<|\eta |<2.1$, and $2.1<|\eta |<2.5$ respectively for all bins in $p_{\mathrm {T}} ^{{\upgamma {}{}}}$. The statistical uncertainty in these efficiencies is negligible, and the total uncertainty is mainly due to differences between the electron and photon efficiencies observed in the simulation.

## Experimental measurement

The purity of the selected candidate events is measured bin by bin in photon $p_{\mathrm {T}} ^{{\upgamma {}{}}}$ and $\eta ^{{\upgamma {}{}}}$. In each bin, a data-based template for the BDT output is defined for the background, and a simulation-based template is defined for the signal. The final purity is estimated using a binned maximum likelihood method [26]:

2 $$\begin{aligned} F(x) = f_{\text {sig}}S(x) + (1-f_{\text {sig}})B(x). \end{aligned}$$

Here *x* is the BDT output, *F*(*x*) denotes the fit template, *S*(*x*) denotes the unity normalized signal template distribution, and *B*(*x*) denotes the unity normalized background template distribution. The $f_{\text {sig}}$ parameter describes the signal purity present in the data and is obtained by maximizing the likelihood, which is equivalent to minimizing the negative of the log-likelihood defined as,

3 $$\begin{aligned} -\log {L(f_{\text {sig}};x_1,x_2,\ldots x_N)} = -\varSigma _N\log {F(x_i|f_{\text {sig}})}. \end{aligned}$$

In the above equation, $L(f_{\text {sig}};x_1,x_2,\ldots x_N)$ is the likelihood function as a function of the $f_{\text {sig}}$ parameter, $x_i$ represent the individual observed values, and *N* represents the total number of data points. The template shape uncertainties are not treated as nuisance parameters, but are characterized using sample experiments as detailed in Sects. 4.1 and 4.2 below.

### Signal templates

Signal templates are obtained using photon+jet simulated events. Because the signal template is obtained from simulation, a data control sample is used to estimate potential differences between data and simulation. Samples of $\mathrm{Z}/{\upgamma {}{}} ^{*}\rightarrow \upmu ^+\upmu ^-{\upgamma {}{}}$ events are obtained by selecting events in which there are two muons and a photon candidate that is produced via final-state radiation from one of the muons. Requiring that the dimuon mass be less than the mass of the on-shell $\mathrm{Z}$ boson allows for the reconstruction of a mass peak in the three-body mass ($m_{\upmu ^+\upmu ^-{\upgamma {}{}}}$) distribution. The sample of events in the peak of the distribution, $80<m_{\upmu ^+\upmu ^-{\upgamma {}{}}}<100$ $\,\text {GeV}$, is enriched with photons, though some background under the peak remains. The remaining background in the BDT distribution is estimated using the sidebands, which are obtained by inverting the $m_{\upmu ^+\upmu ^-{\upgamma {}{}}}$ criteria, and subtracted. The resulting distribution for data photons is then compared to the response in the simulation in the limited range of $p_{\mathrm {T}} ^{{\upgamma {}{}}}$ available. The difference is assigned as a systematic uncertainty in the signal shape for all $p_{\mathrm {T}} ^{{\upgamma {}{}}}$, in separate bins of $\eta ^{{\upgamma {}{}}}$.

### Background templates

The background BDT templates are obtained using a data sideband in pileup-corrected particle-flow photon isolation. Except for the photon isolation constraint, the sideband data is required to pass the same requirements as the signal. Sideband optimization is performed using simulations to select a photon isolation region with sufficient amount of data and minimum correlations between this quantity and the output of the BDT that is used to fit for the final purity. Using a mixture of simulated events containing both dijets and photon+jets, a range of isolation windows are examined. For each bin of $\eta ^{{\upgamma {}{}}}$ and $p_{\mathrm {T}} ^{{\upgamma {}{}}}$, a range of sideband windows are used to generate background templates by varying the candidate photon isolation constraint to an upper bound determined by data set size (nominally 4.5–5$\,\text {GeV}$). Based on the observed data sample size, template shapes are generated randomly from the simulated shapes and then are used to perform a fit to a separate mixture of simulation with a known signal fraction. Based on these generated shapes, the bias between the known signal fraction and the signal fraction from the fit is determined using 500 trials, and the central value of this distribution is taken as the bias induced by the residual correlations. Background shapes are estimated separately for the different pseudorapidity and $p_{\mathrm {T}}$ regions. The uncertainty in the correction for the bias and the difference between the final selected data template and the simulated shape are the systematic uncertainties in the background shape.

### Fit and systematic uncertainties

**Fig. 1:**
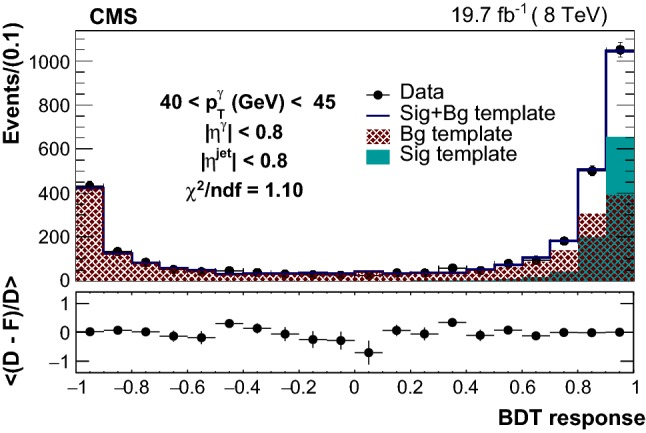
An example fit of candidate boosted-decision-tree distribution with a composite template (blue histogram). The signal (background) template is shown by the green (red) solid (hatched) region. The bottom panel shows the mean of the fit values for 500 templates varied within the signal and background shape uncertainties (F) subtracted from data (D) divided by the data

**Fig. 2:**
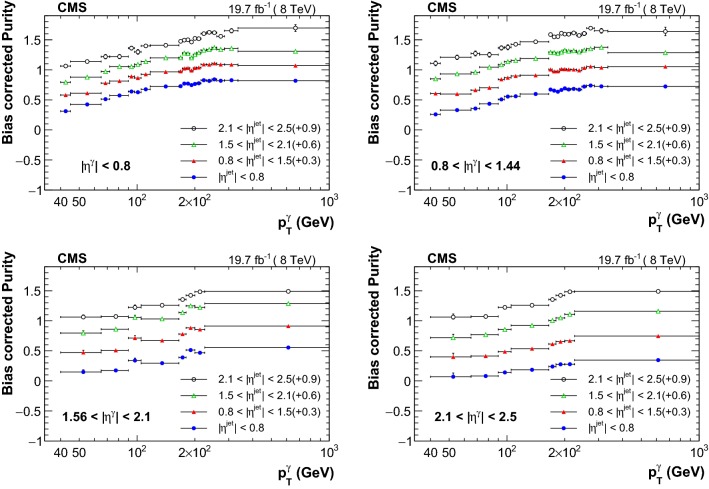
Purity estimates as a function of $p_{\mathrm {T}} ^{\upgamma {}{}}$ for different photon and jet pseudorapidity regions. The values are offset by 0.3, 0.6 and 0.9 for $0.8<|\eta ^{\text {jet}} |<1.5$, $1.5<|\eta ^{\text {jet}} |<2.1$, and $2.1<|\eta ^{\text {jet}} |<2.5$ respectively. The total uncertainties are shown as error bars

In each bin of $|\eta ^{{\upgamma {}{}}} |$, $|\eta ^{\text {jet}} |$, and $p_{\mathrm {T}} ^{{\upgamma {}{}}}$ the purity is estimated by a simultaneous fit to the BDT output using the previously defined signal and background templates. An example fit are shown in Fig. 1. The uncertainty in this measured purity is estimated from sample distributions generated by varying the signal and background fit templates within their respective uncertainties. For the signal template, where the uncertainty contribution is from differences between simulation and detector response, the shapes of sample distributions are obtained by simultaneous variations across different bins of the BDT template. On the other hand, the source of background template shape uncertainty is the data sideband statistical uncertainty, which is uncorrelated across different bins of the BDT distribution. Therefore, the sample distributions for the background template are created by allowing the adjacent bins to vary independently of each other. The purity estimated in each bin and the associated uncertainty is shown in Fig. 2. The signal purity is lower at larger photon rapidities, where the selection criteria are less effective at separating direct photon signals from photons from meson decays because of the smaller opening angle between the daughter photons.

The residual bias caused by correlations is minimized, but not completely eliminated, using the sideband optimization process described in Sect. 4.2. To compensate for this residual bias, a correction is applied based on the estimated bias from the simulation. The correction applied to correct for residual bias in purity decreases as $p_{\mathrm {T}} ^{{\upgamma {}{}}}$ increases. These corrections have associated uncertainties from the size of the simulated data samples and systematic uncertainties of the template shapes. If the bias correction uncertainty is larger than the associated correction, then the correction is not applied, and the amount of bias is taken as an additional systematic uncertainty. The bias-related uncertainty ranges from 0.01 to 4.70% (0.05–10.10%) in the barrel (endcap) region. A summary of the uncertainty in the purity from different sources is provided in Table 1.

**Table 1:** Summary of uncertainties in the estimated purity for photons in the barrel (endcap) region

Sources	Barrel photons (%)	Endcap photons (%)
Statistical	0.5–18.7	0.8–9.2
Signal template shape	0.2–3.7	0.3–7.3
Background template shape	0.4–5.2	1.3–88.7
Residual bias	0.01–4.7	0.05–10.1
Total systematic	0.6–7.8	1.5–89.3

### Unfolding

The cross section measurements are unfolded within the fiducial volume of acceptance and phase space, which are as defined previously in this paper. With the excellent energy resolution of the ECAL, and the width of the selected bins, bin-to-bin migrations are small, but still corrected in the final result. The response matrix is determined from the true generator level $p_{\mathrm {T}} ^{{\upgamma {}{}}}$ and the smeared values obtained from the simulation. The D’Agostini iterative unfolding method, implemented in the RooUnfold [27] package, is used to unfold the detector effects. A systematic uncertainty in this unfolding, due to the input $p_{\mathrm {T}} ^{{\upgamma {}{}}}$ distribution, is obtained by reweighting the input distribution to resemble the spectrum observed in data, reproducing the response matrix, and taking the difference between the unfolded results from the reweighted response matrix to the unreweighted one. The final (small) uncertainty from this procedure is propagated to the final cross section result.

## Comparisons with theory

The measured cross sections are compared with next-to-leading order (NLO) predictions using the modified version of the GamJet [28, 29] package. The recent CJ15 [30] parton distribution functions are used as input to this prediction, and uncertainties are assigned based on the deviation from the 24 pairs of varied PDFs supplied with the CJ15 set. A tolerance factor of 1, assuming that all of the datasets used in the PDF calculation are statistically compatible and the experimental uncertainties are Gaussian, is used for the theoretical prediction. Set II of Bourhis–Fontannaz–Guillet (BFG) [31] fragmentation functions are applied to the matrix element calculations to estimate the photon production via parton fragmentation. Although contributions from fragmentation photons are included in these predictions, an isolation criterion requiring less than 4$\,\text {GeV}$ of hadronic energy within a cone of radius $\varDelta R<0.2$ around the photon direction is utilized, removing a large fraction of them. The central values of the renormalization, fragmentation, and PDF scales are set to $p_{\mathrm {T}} ^{{\upgamma {}{}}}$. The scale uncertainty is quantified by varying each of the scales by factors of 0.5 and 2.0 independently, and the largest variation is taken as the systematic uncertainty. In general, the scale (PDF) uncertainty is dominant in the low (high) photon pseudorapidity bins, with the total uncertainty ranging from 10 to 25% in most cases, and as high as 70% in some $p_{\mathrm {T}} ^{{\upgamma {}{}}}$ bins in the high $|\eta ^{\text {jet}} |$ region.

**Fig. 3:**
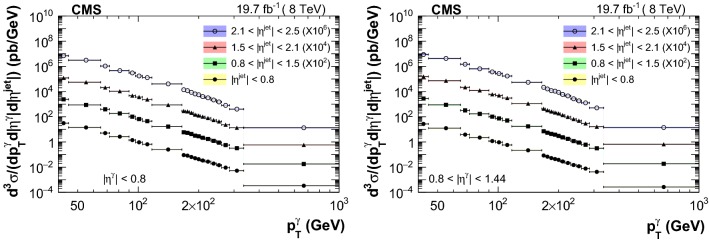
Measured triple-differential cross section distributions as a function of $p_{\mathrm {T}} ^{\upgamma {}{}}$ in different bins of $|\eta ^{\text {jet}} |$ for photons in the barrel region. Note that the distributions are multiplied by a factor of $10^2$, $10^4$ and $10^6$ for $0.8<|\eta ^{\text {jet}} |<1.5$, $1.5<|\eta ^{\text {jet}} |<2.1$, and $2.1<|\eta ^{\text {jet}} |<2.5$ respectively. The statistical (systematic) uncertainties are shown as error bars (color bands)

**Fig. 4:**
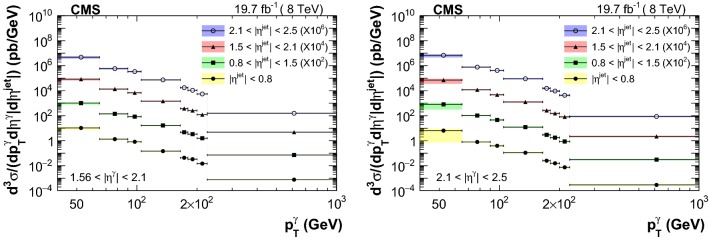
Measured triple-differential cross section distributions as a function of $p_{\mathrm {T}} ^{\upgamma {}{}}$ in different bins of $|\eta ^{\text {jet}} |$ for photons in the endcap region. Note that the distributions are multiplied by a factor of $10^2$, $10^4$ and $10^6$ for $0.8<|\eta ^{\text {jet}} |<1.5$, $1.5<|\eta ^{\text {jet}} |<2.1$, and $2.1<|\eta ^{\text {jet}} |<2.5$ respectively. The statistical (systematic) uncertainties are shown as error bars (color bands)

**Fig. 5:**
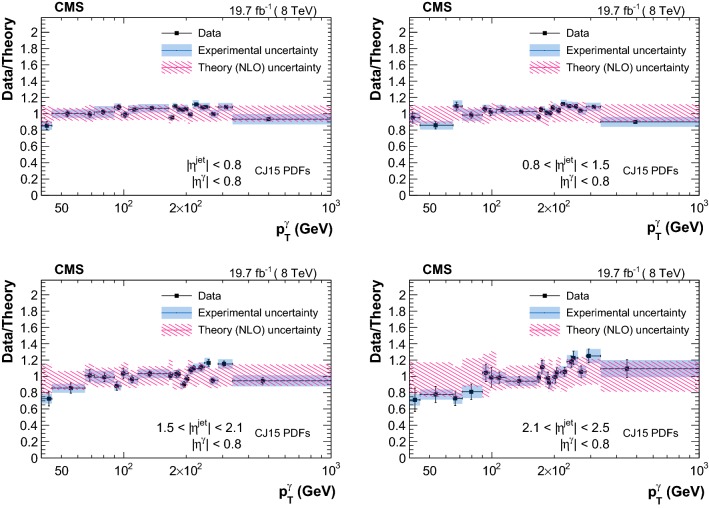
Ratio of triple-differential cross sections as a function of $p_{\mathrm {T}} ^{\upgamma {}{}}$ measured in data over the corresponding GamJet NLO theoretical prediction (obtained with the CJ15 PDFs) in different bins of $|\eta ^{\text {jet}} |$ for $|\eta ^{\upgamma {}{}} |<0.8$. Error bars on the data are statistical uncertainties, and blue bands represent the systematic uncertainties

**Fig. 6:**
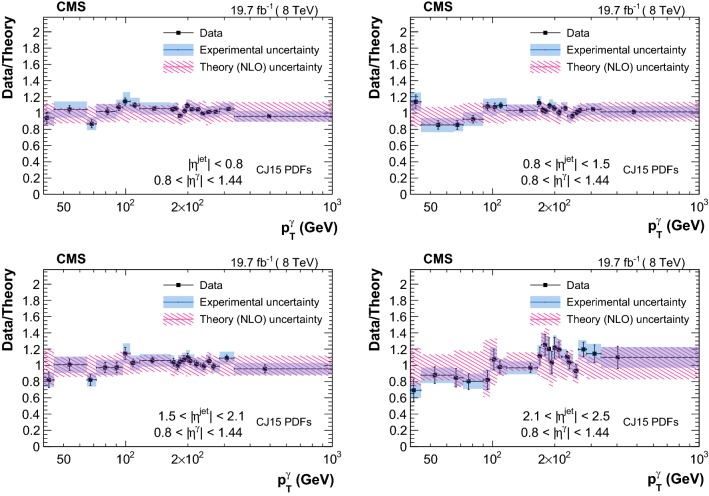
Ratio of triple-differential cross sections as a function of $p_{\mathrm {T}} ^{\upgamma {}{}}$ measured in data over the corresponding GamJet NLO theoretical prediction (obtained with the CJ15 PDFs) in different bins of $|\eta ^{\text {jet}} |$ for $0.80<|\eta ^{\upgamma {}{}} |<1.44$. Error bars on the data are statistical uncertainties, and blue bands represent the systematic uncertainties

**Fig. 7:**
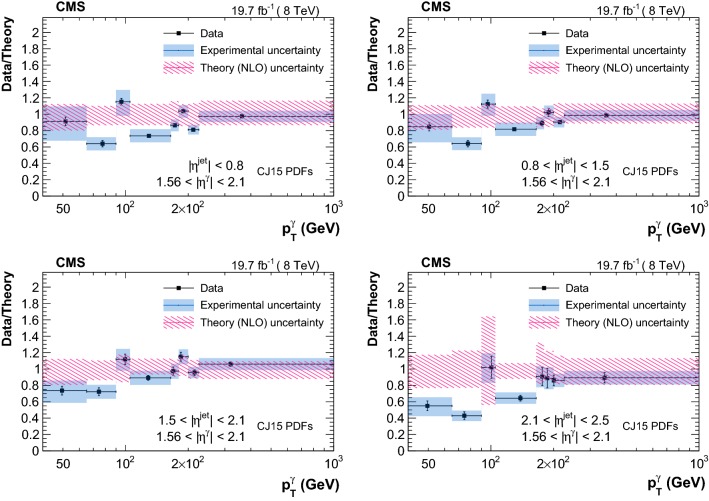
Ratio of triple-differential cross sections as a function of $p_{\mathrm {T}} ^{\upgamma {}{}}$ measured in data over the corresponding GamJet NLO theoretical prediction (obtained with the CJ15 PDFs) in different bins of $|\eta ^{\text {jet}} |$ for $1.56<|\eta ^{\upgamma {}{}} |<2.10$. Error bars on the data are statistical uncertainties, and blue bands represent the systematic uncertainties

**Fig. 8:**
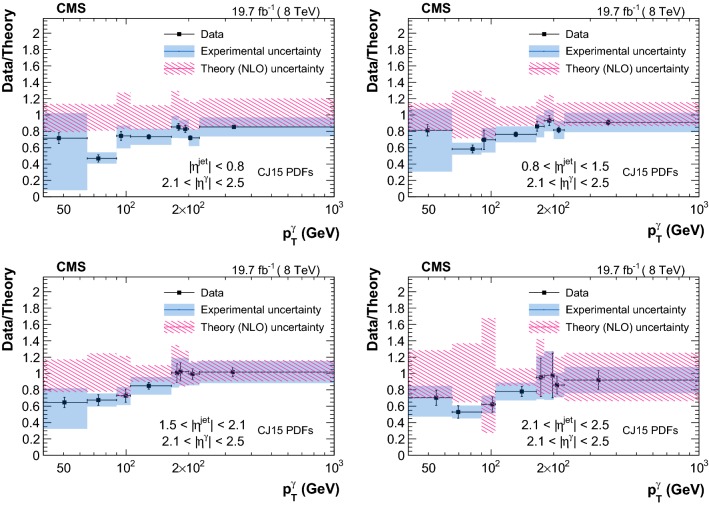
Ratio of triple-differential cross sections as a function of $p_{\mathrm {T}} ^{\upgamma {}{}}$ measured in data over the corresponding GamJet NLO theoretical prediction (obtained with the CJ15 PDFs) in different bins of $|\eta ^{\text {jet}} |$ for $2.1<|\eta ^{\upgamma {}{}} |<2.5$. Error bars on the data are statistical uncertainties, and blue bands represent the systematic uncertainties

The measured triple-differential cross sections are shown in Figs. 3 and 4 . A summary of the uncertainty in the measured cross sections from different sources is reported in Table 2. Comparison between data and theory, along with the respective uncertainties, are provided in Figs. 5, 6, 7 and 8. The measurements are in good agreement with the NLO QCD predictions from GamJet except in the regions of low $p_{\mathrm {T}} ^{{\upgamma {}{}}}$ for endcap photons, where differences of up to 60% are observed between central values of the data and theoretical predictions.

**Table 2:** Summary of the uncertainties in the measured cross section values for photons in the barrel (endcap) region

Sources	Barrel photons (%)	Endcap photons (%)
Statistical	1–20	1–10
Purity	1–9	3–66
Efficiency	1–9	5–11
Luminosity	3	3
Unfolding	0–5	0–1
Total systematic	4–12	6–66

## Summary

Measurements of the triple-differential inclusive isolated-photon+jet cross section were performed as a function of photon transverse momentum ($p_{\mathrm {T}} ^{{\upgamma {}{}}}$), photon pseudorapidity ($\eta ^{{\upgamma {}{}}}$), and jet pseudorapidity ($\eta ^{\text {jet}}$). The measurements were carried out in $\mathrm{p}$ $\mathrm{p}$ collision at $\sqrt{s} = 8\,\text {TeV}$ using 19.7$\,\text {fb}^{-1}$ of data collected by the CMS detector covering a kinematic range of $|\eta ^{{\upgamma {}{}}} |<1.44$ and $1.57<|\eta ^{{\upgamma {}{}}} |<2.50$, $|\eta ^{\text {jet}} |<2.5$, $40< p_{\mathrm {T}} ^{{\upgamma {}{}}}<1000$ $\,\text {GeV}$, and jet transverse momentum, $p_{\mathrm {T}} ^{\text {jet}}$, >25$\,\text {GeV}$. The photon purity was estimated using a combination of templates from data and simulation, based on a multivariate technique. The measured cross sections are in good agreement with the next-to-leading order perturbative quantum chromodynamics (pQCD) prediction, and the experimental uncertainties are comparable or smaller than the theoretical ones. These measured cross sections, in different combinations of photon and jet pseudorapidities, probe pQCD over a wide range of parton momentum fractions. Inclusion of such gluon-sensitive data into the global parton distribution function (PDF) fit analyses has the potential to constrain the gluon PDFs, particularly in the regions where the measured uncertainties are smaller than the uncertainty bands of theoretical predictions.

## Data Availability

This manuscript has no associated data or the data will not be deposited. [Authors’ comment: Release and preservation of data used by the CMS Collaboration as the basis for publications is guided by the CMS policy as written in its document "CMS data preservation, re-use and open access policy" (https://cms-docdb.cern.ch/cgi-bin/PublicDocDB/RetrieveFile?docid=6032&filename=CMSDataPolicyV1.2.pdf&version=2).]
